# Walking around the preferred speed: examination of metabolic, perceptual, spatiotemporal and stability parameters

**DOI:** 10.3389/fphys.2024.1357172

**Published:** 2024-02-09

**Authors:** Lina Majed, Rony Ibrahim, Merilyn Jean Lock, Georges Jabbour

**Affiliations:** ^1^ Exercise Science, Health and Epidemiology Division, College of Health and Life Sciences, Hamad Bin Khalifa University, Doha, Qatar; ^2^ Physical Education Department, College of Education, Qatar University, Doha, Qatar

**Keywords:** gait, perceived exertion, self-selected intensity, stability, fuel oxidation, energy expenditure, stride parameters, exercise

## Abstract

Walking is the most accessible and common type of physical activity. Exercising at one’s self-selected intensity could provide long-term benefits as compared to following prescribed intensities. The aim of this study was to simultaneously examine metabolic, perceptual, spatiotemporal and stability parameters at an absolute 3 km·h^−1^ speed range around the individual preferred walking speed (PWS). Thirty-four young sedentary adults (18 women) volunteered to walk at seven speeds relative to their PWS in 3-min trials interspaced with 3-min rest intervals. Results indicated a significant main effect of speed on all studied variables. While metabolic, perceptual and spatiotemporal values were sensitive to the smallest change in speed (i.e., 0.5 km·h^−1^), a significant increase in the rate of carbohydrate oxidation and decrease in %fat oxidation were only observed at speeds above PWS. Results also revealed significantly higher coefficients of variation for stride characteristics at speeds below PWS only. Moreover, analyses of best fit models showed a quadratic relationship between most variables and speed, with the exceptions of metabolic cost of transport, rating of perceived exertion and stride duration that changed exponentially with speed. PWS coincided with optimized mechanical efficiency, fuel oxidation and gait stability. This indicated that walking below PWS decreased both mechanical efficiency and stability of gait, while walking above PWS increased carbohydrate oxidation. Those factors seem to play an important role as determinants of PWS. We suggest that walking at PWS may provide benefits in terms of fat oxidation while optimizing gait stability.

## 1 Introduction

Walking is the most natural, accessible, and common daily lifestyle activity. Although walking is viewed as a simple skill, it is considered as a complex behavior resulting from a large number of components (e.g., nervous and sensory systems, muscles, bones, joints) interacting to produce a stable pattern (Diedrich and Warren, 1995). While most people can walk at speeds reaching up to about 9 km·h^−1^, they naturally choose to walk at a typical speed (i.e., around 4.5 km·h^−1^) known as the preferred walking speed (PWS) (Bohannon and Andrews, 2011). From a dynamic perspective, walking at PWS is a behavior known to possess properties of an attractor, in which any small change in the speed (i.e., control parameter) in either direction would result in loss of stability (Diedrich and Warren, 1995). According to self-optimization theories (Sparrow and Newell, 1998; Alexander, 2002), Humans naturally tend to adopt behaviors that minimize metabolic energy cost and other cost functions (e.g., mechanical, perceptual, cognitive). The relationship between walking speed and metabolic cost of transport has long been described as a U-shaped curve (Ralston, 1958; Zarrugh, Todd and Ralston, 1974; Margaria, 1976), indicating an optimum at PWS. Several studies have attempted to identify determinants of preferred behaviors or preferred intensities. Indeed, walking at PWS has been recognized to optimize not only energy cost, but also substrate utilization (Willis et al., 2005), biomechanical parameters (Saibene and Minetti, 2003), perceived exertion (Willis et al., 2005; Godsiff et al., 2018), cognitive function (Abernethy et al., 2002), and gait stability (Jordan et al., 2007). For instance, Willis et al. (2005) concluded that healthy young adults choose a PWS that minimizes carbohydrate oxidation rate. While there seems to be several competing factors being optimized at PWS (Fernández Menéndez et al., 2019), determinants of the spontaneous adoption of PWS are still a topic of debate.

From an evolutionary perspective, people are instinctively inclined to optimize their physical exertion when there is no need obtain food (Eaton and Eaton, 2003). In today’s modern societies, meeting minimal physical activity recommendations for health requires a cognitive effort (Peters et al., 2002), and a certain level of motivation that becomes even more challenging when exercise is recommended or performed at higher intensities (Perri et al., 2002; Parfitt and Hughes, 2009; Ekkekakis et al., 2011). From a behavioral perspective, exercising at one’s self-selected or preferred intensity may contribute to greater health benefits in the long-term (Ekkekakis et al., 2008; Williams, 2008). Walking is a major activity recommendation for health and disease prevention (Carnethon et al., 2009; Williams et al., 2014), therefore more focus is needed to better understand the multiple acute responses when walking at or around the preferred intensity as a primary mode of exercise, especially in sedentary individuals.

Specific physical activity guidelines or exercise recommendations usually relate to frequency, duration, and intensity (e.g., 40%–59% $\dot{V}O_{2}$ reserve or RPE of 12–13) that might not be as accessible or as effective for changing behavior in inexperienced populations compared to just “moving” the way they prefer. For example, previous experimental studies have found that low-active, overweight adults volitionally completed more minutes per week of self-paced walking compared to walking regulated using prescribed heart rates (Williams et al., 2015).

Walking parameters have been analyzed at and around PWS, and studies have investigated how changes in walking speed affect gait and other acute responses. However, most studies have used a range of set absolute speeds for all participants (Willis et al., 2005; Fernández Menéndez et al., 2019), or expressed speed as a percentage of PWS (Jordan et al., 2007; Chung and Wang, 2010), or even defined speed as slow, self-selected and fast (Haight et al., 2014). The present study aims to examine metabolic, perceptual, spatiotemporal and gait stability parameters at an absolute range of speeds (3 km·h^−1^) around each participant’s own PWS. We hypothesized that sedentary young adults choose to walk at speeds that optimize certain cost function (e.g., metabolic cost, perceived exertion, stability), while providing benefits in terms of fuel oxidation (Willis et al., 2005). Evidence of optimization at PWS is expected to be seen following one of two criteria. First, the minimum or maximum of a quadratic relationship between the studied variable and speed should coincide with PWS indicating an optimum. Second, with increasing speeds evidence of a deflection in values of the studied variable should happen at PWS. In addition to examining determinants of PWS, the present study further attempts to understand potential benefits of walking at or around the PWS, rather than at intensities relative to the maximum or reserve heart rate or oxygen consumption.

## 2 Materials and methods

### 2.1 Participants

Thirty-four healthy volunteers (18 women and 16 men) aged between 18 and 26 years old took part in the study that was approved by the local Ethics Committee (QU-IRB 1696-E/22). Participants were recruited from the student body via word of mouth and through advertisement (i.e., flyers) shared at various locations of the university campus. Participants were sedentary (i.e., low physical activity level), non-smokers and did not present any past or present disease (e.g., neurological, cardiovascular, respiratory, metabolic diseases), or other health complications (e.g., injury, motor impairment) that might interfere with their ability to walk normally. None of them was taking any medication and their body mass did not vary more than 2.5 kg in the last 3 months. Participants’ physical characteristics are shown in Table 1. Prior to the experiment, a written informed consent was obtained from each volunteer in accordance with the Declaration of Helsinki.

**TABLE 1:** Participants’ physical characteristics and preferred walking speeds (PWS). Data are presented as mean (standard deviation).

	Female	Male	All
Sample size	18	16	34
Age (years)	21.67 (1.91)	23.49 (2.28)*	22.52 (2.26)
Body height (cm)	158.06 (4.57)	174.31 (7.15)**	165.71 (10.09)
Body mass (kg)	57.42 (14.16)	72.89 (18.76)*	64.70 (18.02)
Body mass index	22.90 (5.08)	23.81 (5.04)	22.52 (2.26)
Preferred walking speed (m∙s^−1^)	1.12 (0.12)	1.16 (0.19)	1.14 (0.16)
Preferred walking speed (km∙h^−1^)	4.03 (0.43)	4.18 (0.68)	4.10 (0.58)

*p < 0.05, **p < 0.01.

### 2.2 Protocol

Participants reported once to the laboratory after a 10–12 h overnight fast, wearing comfortable sports outfits and footwear. They were instructed to refrain from exercise and caffeinated products in the 24 h preceding the experiment. All tests were conducted in the morning (between 9a.m. and 12p.m.) and under similar environmental conditions (i.e., 55% relative humidity and 22°C).

After an initial physical activity screening using the short-form of the International Physical Activity Questionnaire (IPAQ-SF, Craig et al., 2003), body mass and height were collected (i.e., body mass and body height). A 10-min familiarization with treadmill was carried out (Meyer et al., 2019) to ensure that participants’ gait was representative of treadmill walking and a stable performance was reached. The protocol was then divided into two consecutive testing phases lasting approximately 2 h.

#### 2.2.1 Determination of the preferred walking speed

A standardized treadmill test was used to determine the individual preferred walking speed (PWS) (Jordan et al., 2007; Dal et al., 2010). Participants, blind to the displayed digital speed, started by walking at a slow pace of 2 km⋅h^−1^, after which increments of 0.1 km⋅h^−1^ followed every 10 s until the most comfortable speed was reported. At that stage, the treadmill’s speed increased by 1.5 km⋅h^−1^ and speed decrements of 0.1 km⋅h^−1^ followed every 10 s until participants reported once again reaching their PWS. This procedure was repeated three times with a 3-min rest interval between trials. The PWS was then calculated as the mean of the six reported speed values.

#### 2.2.2 Walking test

The second experimental phase was performed after a 10-min seated rest and aimed to collect physiological, perceptual, and spatiotemporal parameters for walking at different absolute speed levels around the PWS. The test consisted in seven 3-min walking trials at speeds relative to the PWS (i.e., in the following order: PWS-1.5 km⋅h^−1^, PWS-1 km⋅h^−1^, PWS-0.5 km⋅h^−1^, PWS, PWS+0.5 km⋅h^−1^, PWS+1 km⋅h^−1^ and PWS+1.5 km⋅h^−1^). Speed trials were interspaced by a 3-min resting period to allow enough recovery and avoid any fatigue effect. Prior to testing, participants were instructed on how to report their rating of perceived exertion (RPE) by raising their index finger to indicate their score while the experimenter read the scale up from 6 to 20 (Borg, 1973). Finally, participants were fitted with the gas analyzer and heart rate monitor.

### 2.3 Apparatus

All walking trials were performed on a motorized treadmill (Valiant 2 CPET, Lode, the Netherlands) set at a gradient of 0%. A metabolic gas analyzer was used to collect respiratory and gas exchange data (Metalyzer 3B with MetaSoft Studio software, Cortex Medical, Germany). A standardized calibration procedure was undertaken before each test according to the manufacturer’s instructions for ambient air, reference gases of known concentrations (with a gas bottle) and airflow volume (with a 3-L syringe) (Medbo et al., 2002). A synchronized heart rate polar chest belt (Polar, Kempele, Finland) and a 6–20 Borg scale (Borg, 1973) were used for heart rate data and ratings of perceived exertion (i.e., 6 for no exertion at all, 20 for maximal exertion).

A high-definition camcorder (CCD-TRV66, Sony, Japan) recorded the intermediate 1-min interval of each of the seven 3-min walking trials at a frequency of 25 Hz. The camera was set at a standard position perpendicular to the left mid-point of the treadmill’s long axis at a 2-m distance. Participants were equipped with two reflective markers on their left heel (i.e., calcaneus) and toe (i.e., second metatarsal head) to allow the detection of heel-strike and toe-off events. A video-based analysis and modeling software were used to digitalize and compute spatiotemporal data (Tracker 4.91, Open Source Physics; Brown, 2015).

### 2.4 Data processing

#### 2.4.1 Physiological and perceptual data

Oxygen consumption ( $\dot{V}O_{2}$ , L⋅min^−1^), carbon dioxide production ( $\dot{V}CO_{2}$ , L⋅min^−1^), minute ventilation ( $\dot{V}$ E, L⋅min^−1^) and heart rate (HR, beats⋅min^−1^) were continuously recorded during the seven walking trials. Respiratory exchange ratio (RER) was computed as the ratio between $\dot{V}CO_{2}$ and $\dot{V}O_{2}$ . Mean values were determined at the last minute of each trial when a steady state was reached. The relative oxygen consumption (r $\dot{V}O_{2}$ , mL⋅kg^−1^⋅min^−1^) was calculated from the individual body mass values. The net relative $\dot{V}O_{2}$ per distance traveled was computed to obtain the metabolic cost of transport (MCT, mL⋅kg^−1^⋅km^−1^, di Prampero, 1986) with speed expressed in km⋅h^−1^ and a resting $\dot{V}O_{2}$ value set at 3.5 mL⋅kg^−1^⋅min^−1^ (Medbo and Tabata, 1989). Steady state $\dot{V}O_{2}$ and $\dot{V}CO_{2}$ values were used to compute the energy expenditure (EE, kcal⋅min^−1^) according to Brouwer (1957). Percent fat oxidation (%Fat) was calculated in relation to the mean steady state non-protein RER following McGilvery and Goldstein’s (1983) method. The rates of fat and carbohydrate (CHO) oxidation were calculated in g. min^−1^ according to the non-protein RER (Peronnet and Massicotte, 1991). The values were then expressed in gA78Fkg^−1^⋅h^−1^ relatively to body mass.

Gross mechanical efficiency (ME) was computed based on Lafortuna et al.’s (2006) equation, as the ratio of work expressed as the absolute speed in km⋅h^−1^ and the rate of energy consumed (E, W. min^−1^) that was in turn calculated according to Garby and Astrup (1987). RPE values were collected exactly 20 s before the end of each 3-min walking trial when participants raised the index finger to indicate their RPE value as the experimenter read up the scale.

#### 2.4.2 Movement-related data

Spatiotemporal gait parameters were obtained by examining 15 full strides recorded in the middle of each trial (i.e., between the first and second minutes) (Hansen et al., 2017). For each stride, the exact timings (i.e., frames) at which the left heel touched the ground (i.e., heel strike) and the left toe left the ground (i.e., toe-off) were recorded by an experienced researcher. [1] Stride duration (SD, s) represented the time between two consecutive left heel strikes, [2] support phase duration (SPD, s) was considered as the time elapsed between left heel strike and the consecutive left toe off within the same stride, [3] stride length (SL, m⋅stride^−1^) was computed as the product of the SD and the corresponding trial’s speed (in m⋅s^−1^) and [4] stride frequency (SF, stride⋅s^−1^) was calculated as the ratio between the corresponding speed (in m⋅s^−1^) and SL. Furthermore, the coefficient of variation (CoV) for SD, SPD, SF and SL were calculated as the ratio between the standard deviation and the mean of values relative to the 15 individual strides collected at each speed trial. The latter was meant to account for dynamic stability in gait as done by Fischer et al. (2021). Speed is presented in km⋅h^−1^ in the text for comparison purposes.

### 2.5 Statistical analysis

The normality of all data sets was verified using Shapiro-Wilk’s tests and by visual inspection of Q-Q plots. Independent *t*-tests were used to compare physical characteristics and PWS between male and female groups. Mixed analyses of variances (ANOVAs) were performed to examine main and interaction effects of Speed (7 repeated measures in km⋅h^−1^: PWS-1.5, PWS-1, PWS-0.5, PWS, PWS+0.5, PWS+1 and PWS+1.5) and Gender (between-subject factor) on all physiological (i.e., r $\dot{V}O_{2}$ , EE, MCT, ME, HR, $\dot{V}$ E, %Fat, Fat, CHO), perceptual (i.e., RPE) and movement-related (i.e., SF, SL, SD, SPD, and their CoVs) variables. Assumptions of homogeneity and sphericity were verified and a Huynh-Feldt procedure was used to adjust the significance of *p*-values and F (degrees of freedom) to control for possible violations (Huynh and Feldt, 1970). Analyses were completed when needed with pairwise *post hoc* comparisons with Bonferroni adjustments to the significance level. Best fit regression models were performed to examine the relationship between each dependent variable and relative walking speeds. The best fit model was considered only if the regression model was significant (ANOVA results, *p* < 0.05) and presented the highest adjusted coefficient of determination (*R* ^2^) value compared to other models. When the adjusted *R* ^2^ values were similar, the model with the highest F value was retained. All data are presented as mean and standard deviation. Statistical analyses were performed using SPSS (IBM, version 28) with a level of significance set at *p* < 0.05.

## 3 Results

### 3.1 Physical characteristics

PWS of the male and female groups did not differ significantly (*p* > 0.050, Table 1). Male participants were on average 1.82 years older than female participants [*t* (32) = 2.535, *p* = 0.016], while no significant differences in their body mass index (BMI) was present (Table 1). Body mass and body height were significantly higher for the male group as compared to the female group [*t* (32) = 2.732, *p* = 0.010; *t* (32) = 7.989, *p* < 0.001, respectively].

### 3.2 Physiological and perceptual data

**FIGURE 1:**
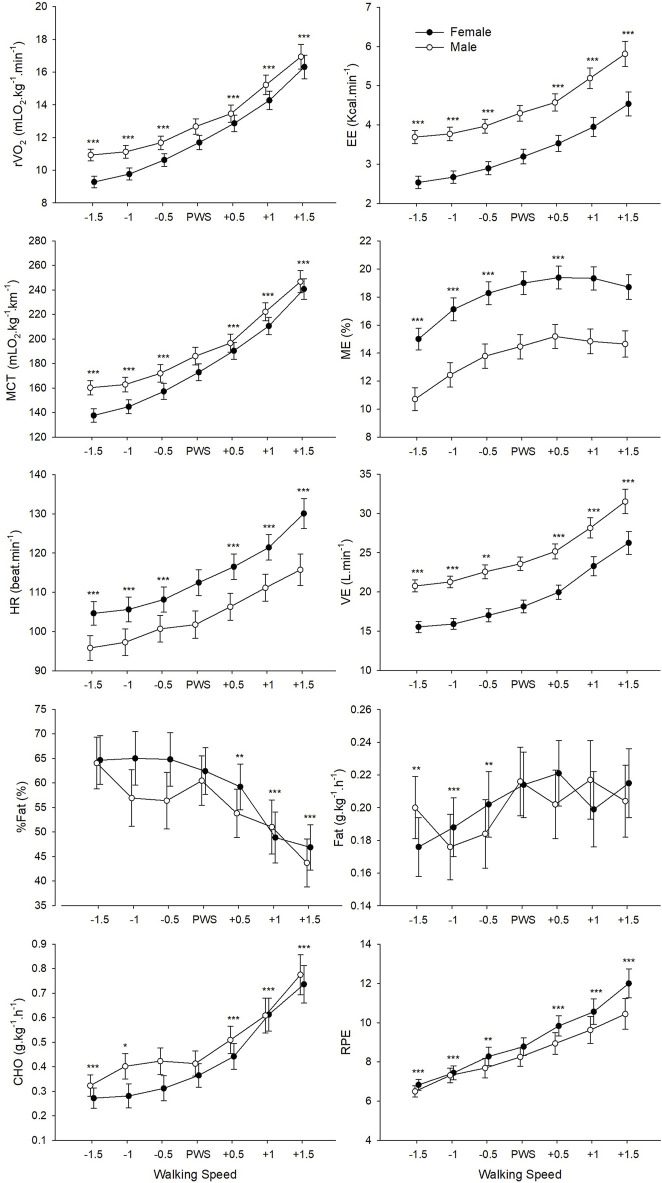
Mean values of physiological and perceptual variables for male and female groups according to the seven tested speeds relative to the preferred walking speed (PWS) in km·h^−1^. r $\dot{V}O_{2}$ , relative oxygen consumption; EE, energy expenditure; MCT, metabolic cost of transport; ME, mechanical efficiency; HR, heart rate; $\dot{V}$ E, minute ventilation, %Fat, percent fat oxidation; Fat/CHO, rate of fat/carbohydrate oxidation relative to body weight; RPE, rating of perceived exertion. Error bars represent the standard error. Significant speed-related differences are presented in comparison to PWS, *p < 0.05, **p < 0.01, ***p < 0.001.

ANOVAs revealed a significant main effect of Speed on all physiological data and RPE (Table 2; Figure 1). Post-hoc pairwise comparisons made relative to PWS are displayed in detail in Figure 1. In general, values of most variables (i.e., r $\dot{V}O_{2}$ , EE, MCT, HR, $\dot{V}$ E, RPE) changed significantly at every speed increment and presented significant differences between all speeds. However, the significant increase in ME values with speeds was only seen between PWS-1.5 and PWS+0.5 km⋅h^−1^ after which no further increases were noted. Moreover, %Fat values did not change with speed until PWS+0.5 km⋅h^−1^ where a significant decline was revealed with increasing speeds (Figure 1). Similarly, the rate of Fat oxidation increased significantly with speed until PWS then stabilized, while the opposite was found for the rate of CHO oxidation that increased significantly only at speeds higher than PWS. ANOVAs also revealed a significant main effect of gender on EE, ME, HR and $\dot{V}$ E (Table 2). Although male participants had significantly higher EE and $\dot{V}$ E values as compared to female participants, they presented significantly lower ME and HR (Figure 1). Significant interaction effects were also noted for MCT and HR, indicating no significant gender differences for MCT at speeds higher than PWS-1 km⋅h^−1^ and for HR at speeds lower than PWS (*p* > 0.05).

**TABLE 2:** ANOVAs’ results for the effect of speed and gender on physiological and perceptual variables.

	Tested effect	F (df_1_,df_2_)	η^2^	*p*
r $\dot{V}O_{2}$ (mL⋅kg^−1^⋅min^−1^)	Speed	F (2.110,67.515) = 282.626	0.898	<0.001
	Gender	F (1.32) = 2.386	0.069	0.132
	Speed × Gender	F (2.110,67.515) = 1.766	0.052	0.177
EE (kcal⋅min^−1^)	Speed	F (1.788,57.212) = 205.622	0.865	<0.001
	Gender	F (1.32) = 15.425	0.325	<0.001
	Speed × Gender	F (1.788,57.212) = 0.683	0.021	0.493
MCT (mL⋅kg^−1^⋅km^−1^)	Speed	F (2.959,94.701) = 407.584	0.927	<0.001
	Gender	F (1.32) = 2.023	0.059	0.165
	Speed × Gender	F (2.959,94.701) = 3.054	0.087	0.033
ME (%)	Speed	F (2.076,66.429) = 61.959	0.659	<0.001
	Gender	F (1.32) = 14.841	0.317	<0.001
	Speed × Gender	F (2.076,66.429) = 0.289	0.009	0.758
HR (beats⋅min^−1^)	Speed	F (2.061,65.948) = 175.487	0.846	<0.001
	Gender	F (1.32) = 4.661	0.127	0.038
	Speed × Gender	F (2.061,65.948) = 3.208	0.091	0.045
$\dot{V}$ _E_ (L⋅min^−1^)	Speed	F (2.656,85.003) = 116.322	0.784	<0.001
	Gender	F (1.32) = 17.507	0.354	<0.001
	Speed × Gender	F (2.656,85.003) = 0.088	0.003	0.954
%Fat (%)	Speed	F (4.150,132.784) = 11.683	0.267	<0.001
	Gender	F (1.32) = 0.337	0.010	0.566
	Speed × Gender	F (4.150,132.784) = 0.924	0.028	0.455
Fat (g⋅kg^−1^⋅h^−1^)	Speed	F (3.945,126.253) = 2.876	0.082	0.026
	Gender	F (1.32) = 0.008	0.000	0.929
	Speed × Gender	F (3.945,126.253) = 1.186	0.036	0.320
CHO (g⋅kg^−1^⋅h^−1^)	Speed	F (3.018,96.561) = 42.697	0.572	<0.001
	Gender	F (1.32) = 0.861	0.026	0.360
	Speed × Gender	F (3.018,96.561) = 0.714	0.022	0.546
RPE	Speed	F (1.925,61.585) = 55.077	0.633	<0.001
	Gender	F (1.32) = 1.249	0.038	0.272
	Speed × Gender	F (1.925,61.585) = 1.211	0.036	0.304

r $\dot{V}O_{2}$ , relative oxygen consumption; EE, energy expenditure; MCT, metabolic cost of transport; ME, mechanical efficiency; HR, heart rate; $\dot{V}$ _E_, minute ventilation; %Fat, percent fat oxidation; Fat/CHO, rate of fat/carbohydrate oxidation relative to body mass; RPE, rating of perceived exertion.

### 3.3 Movement-related data

**FIGURE 2:**
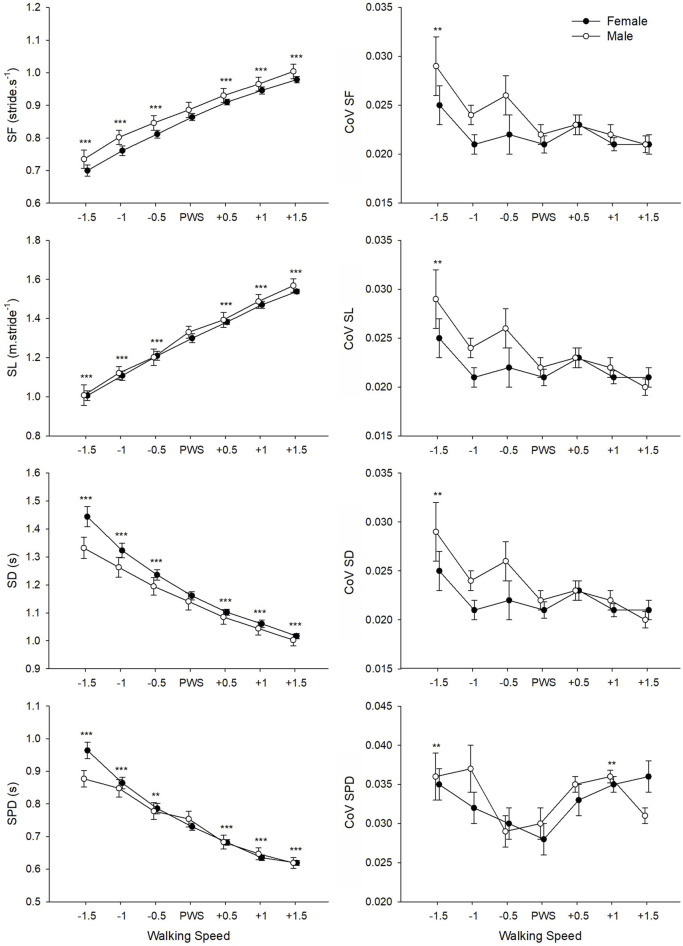
Mean values of movement-related variables for male and female groups according to the seven tested speeds relative to the preferred walking speed (PWS) in km·h^−1^. SF, stride frequency; SL, stride length; SD, stride duration; SPD, support phase duration; CoV, coefficient of variation. Error bars represent the standard error. Significant speed-related differences are presented in comparison to PWS, ^*^ *p* < 0.05, ^**^ *p* < 0.01, ^***^ *p* < 0.001.

ANOVAs found a significant main effect of Speed on all studied movement-related variables (Table 3). For SF, SL, SD, and SPD, a significant change in values was observed at each speed increment (Figure 2). CoV values for SF, SL, and SD presented a similar pattern of change with speed, where values were significantly higher at the lowest tested speed as compared to the four highest speeds (including PWS). The CoV for SPD presented the lowest values at PWS-0.5 km⋅h^−1^ and PWS as compared to lower (i.e., PWS-1.5 km⋅h^−1^) and higher (i.e., PWS+1 km⋅h^−1^) speeds. While no Gender effect appeared in the analyses, an interaction was found on SD and SPD indicating convergence of male and female values at higher speed values.

**TABLE 3:** ANOVAs’ results for the effect of speed and gender on movement related variables.

	Tested effect	F (df1,df2)	η^2^	*p*
SF (stride⋅s^−1^)	Speed	F (2.127,68.071) = 653.731	0.953	<0.001
	Gender	F (1.32) = 1.345	0.040	0.255
	Speed × Gender	F (2.127,68.071) = 1.162	0.035	0.321
SL (m⋅stride^−1^)	Speed	F (2.656, 84.995) = 482.361	0.938	<0.001
	Gender	F (1.32) = 0.131	0.004	0.720
	Speed × Gender	F (2.656,84.995) = 0.619	0.019	0.585
SD (s)	Speed	F (1.747,55.892) = 239.431	0.882	<0.001
	Gender	F (1.32) = 1.615	0.048	0.213
	Speed × Gender	F (1.747,55.892) = 4.229	0.117	0.024
SPD (s)	Speed	F (3.005,96.175) = 215.900	0.871	<0.001
	Gender	F (1.32) = 0.220	0.007	0.642
	Speed × Gender	F (3.005,96.175) = 5.587	0.149	0.001
CoV SF	Speed	F (3.968,126.981) = 4.649	0.127	0.002
	Gender	F (1.32) = 2.578	0.075	0.118
	Speed × Gender	F (3.968,126.981) = 0.741	0.023	0.565
CoV SL	Speed	F (3.950,126.392) = 4.763	0.130	0.001
	Gender	F (1.32) = 2.557	0.074	0.120
	Speed × Gender	F (3.950,126.392) = 0.824	0.025	0.511
CoV SD	Speed	F (3.950, 126.392) = 4.763	0.130	0.001
	Gender	F (1.32) = 2.557	0.074	0.120
	Speed × Gender	F (3.950, 126.392) = 0.824	0.025	0.511
CoV SPD	Speed	F (5.055,161.775) = 4.447	0.122	<0.001
	Gender	F (1.32) = 0.146	0.005	0.705
	Speed × Gender	F (5.055,161.775) = 1.710	0.051	0.134

SD, stride duration; SPD, support phase duration; SF, stride frequency; SL, stride length; CoV, coefficient of variation.

### 3.4 Regressions: best fit model

**FIGURE 3:**
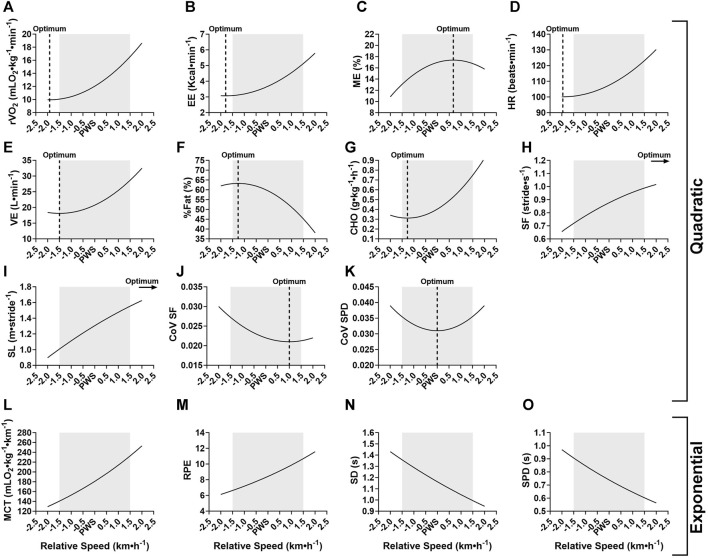
Visual representation of the best fit significant models for tested variables at speeds relative to the preferred walking speed (PWS) in km·h^−1^. **(A)** r $\dot{V}O_{2}$ , relative oxygen consumption; **(B)** EE, energy expenditure; **(C)** ME, mechanical efficiency; **(D)** HR, heart rate; **(E)** $\dot{V}$ E, minute ventilation, **(F)** %Fat, percent fat oxidation; **(G)** CHO, rate of carbohydrate oxidation relative to body weight; **(H)** SF: stride frequency, **(I)** SL, stride length; **(J)** CoV SF, coefficient of variation of the stride frequency (the same function applies to the CoV stride length and stride duration), **(K)** CoV SPD, coefficient of variation of the support phase duration, **(L)** MCT, metabolic cost of transport; **(M)** RPE, rating of perceived exertion; **(N)** SD, stride duration; **(O)** SPD, support phase duration. The light grey area represents the tested speed range. For quadratic models, the optimum (i.e., vertex) is represented with a vertical dashed line.

The relationship between variables and relative walking speed (i.e., PWS-1.5 to +1.5 km⋅h^−1^) was further investigated. This analysis was done by examining the overall best fit regression model for each variable with data from female and male groups pooled together. Results showed that the relationship between most of the studied variables and speed was best described as quadratic (Table 4), with the exceptions of MCT, RPE, SD and SPD, all of which were best described with an exponential model. The rate of fat oxidation was not fitted significantly to any of the studied models (i.e., linear, quadratic, exponential). Adjusted coefficients of determination (*R* ^2^) varied from 3.1% to 69.4%, revealing a higher predictive power of speed for spatiotemporal variables as well as relative oxygen consumption and MCT. Figure 3 offers a better visualization of the actual best fit models for each studied variable. The optimum of quadratic functions is indicated by a dashed vertical line to point to the calculated vertex x-coordinate (i.e., speed).

**TABLE 4:** Best fit regression models for all tested variables in relation to relative walking speeds.

	Best fit model	Adj *R* ^2^	SEE	F (2, 235)	Parameter estimates		
Variables					Constant	b1	b2
r $\dot{V}$ O_2_ (mL⋅kg^−1^⋅min^−1^)	Quadratic	0.525	2.108	131.962***	12.035	2.165	0.566
EE (kcal⋅min^−1^)	Quadratic	0.311	1.043	53.089***	3.678	0.677	0.189
MCT (mL⋅kg^−1^⋅km^−1^)	Exponential	0.592	1.140	344.444***	180.894	0.168	
ME (%)	Quadratic	0.109	4.064	15.515***	16.968	1.234	−0.917
HR (beats⋅min^−1^)	Quadratic	0.215	14.544	33.430***	107.487	7.527	1.922
$\dot{V}$ _E_ (L⋅min^−1^)	Quadratic	0.360	4.852	67.714***	20.747	3.516	1.176
%Fat (%)	Quadratic	0.076	20.994	10.803***	59.482	−5.984	−2.367
Fat (g⋅kg^−1^⋅h^−1^)	ns	ns	ns	ns	ns	ns	
CHO (g⋅kg^−1^⋅h^−1^)	Quadratic	0.293	0.236	50.086***	0.404	0.145	0.057
RPE	Exponential	0.322	0.231	113.330***	8.419	0.159	
SF (stride⋅s^−1^)	Quadratic	0.607	0.073	183.814***	0.876	0.090	−0.010
SL (m⋅stride^−1^)	Quadratic	0.694	0.121	270.322***	1.305	0.182	−0.011
SD (s)	Exponential	0.601	0.085	358.304***	1.162	−0.104	
SPD (s)	Exponential	0.654	0.099	448.427***	0.739	−0.136	
CoV SF	Quadratic	0.059	0.006	8.454***	0.022	−0.002	0.001
CoV SL	Quadratic	0.060	0.006	8.560***	0.022	−0.002	0.001
CoV SD	Quadratic	0.060	0.006	8.560***	0.022	−0.002	0.001
CoV SPD	Quadratic	0.031	0.008	4.843**	0.031	−0.000	0.002

Adj, adjusted; SEE, standard error of estimate; r $\dot{V}O_{2}$ , relative oxygen consumption; EE, energy expenditure; MCT, metabolic cost of transport; ME, mechanical efficiency; HR, heart rate; $\dot{V}$ E, minute ventilation; %Fat, percent fat oxidation; Fat/CHO, rate of fat/carbohydrate oxidation relative to body weight; RPE, rating of perceived exertion; SF, stride frequency; SL, stride length; SD, stride duration; SPD, support phase duration; CoV, coefficient of variation. Significant model fit, **p < 0.01, ***p < 0.001.

## 4 Discussion

The present study investigated metabolic, perceptual, spatiotemporal and stability parameters when walking at a 3 km·h^−1^ range around the individual preferred walking speed (PWS). Main findings indicate that sedentary young adults do not select their PWS based on principles of minimization of metabolic cost (Ralston, 1958; Zarrugh, Todd and Ralston, 1974; Margaria, 1976) or based on perceived exertion (Willis et al., 2005; Fernández Menéndez et al., 2019). Participants preferred to walk at a speed that optimizes fuel oxidation (Willis et al., 2005), mechanical efficiency and gait stability, which confirms in part our hypothesis. Indeed, it seems that several competing factors are optimized at PWS (Fernández Menéndez et al., 2019). However, present results could not confirm the well-established U-shaped relationship between metabolic cost of transport and speed (Ralston, 1958; Zarrugh, Todd and Ralston, 1974; Margaria, 1976), which is in line with a more recent study (Willis et al., 2005). Our results support those demonstrating that PWS does not occur at a minimum energy cost (Godsiff et al., 2018). The absolute speed range considered in the current study (i.e., 0.5 km·h^−1^ increments from PWS-1.5 to PWS+1.5 km·h^−1^) differed according to each participant’s own PWS, which was not the case in previous studies examining the relationship between metabolic and perceptual responses to a set absolute speed continuum for all participants (Willis et al., 2005). Although this might make comparisons harder, factors being optimized at PWS are more easily identified when individual PWS is considered.

The average PWS of the present sample was 4.1 (±0.58) km·h^−1^ which is in accordance with previous studies in Western (Bohannon and Andrews, 2011; Bohannon and Wang, 2019) and Arab populations (Majed et al., 2020; 2022). A first result indicated that walking speed significantly affected all studied metabolic, perceptual, spatiotemporal and stability parameters (Tables 2, 3). Participants were sensitive to the smallest change in speed (i.e., 0.5 km·h^−1^) around PWS, which was also revealed by significant changes in the subjective perception of effort (i.e., RPE). Increasing speeds around PWS was linked to an increase in relative oxygen consumption, energy expenditure, metabolic cost of transport, heart rate, and RPE (Figure 1). Interestingly, certain parameters presented significant speed-related changes only below PWS or above PWS. For instance, values of mechanical efficiency and fat oxidation rate seem to stabilize close to PWS after initially increasing with speed. Moreover, a clear decline in %fat oxidation and a rise in carbohydrate oxidation rate are seen at speeds above PWS only, indicating a possible shift of substrate contribution to energy production (Willis et al., 2005). These important observations indicating a deflection point at or close to PWS in mechanical efficiency and substrate oxidation could be interpreted as potential determinants of PWS.

Findings indicate that participants choose to walk at a speed that not only optimizes mechanical efficiency, but also minimizes reliance on carbohydrate to potentially avoid fatigue (Willis et al., 2005). However, it remains unclear whether walking at PWS corresponds to the range of maximal rate of fat oxidation (Fat_max_). Previous reports have indicated that the maximal rate of fat oxidation is expected to occur at low or moderate intensities around approximately 36%–65% $\dot{V}$ O_2max_ (Achten and Jeukendrup, 2004). In a recent study examining changes in the rate of fat oxidation during walking in sedentary young men (29.3 ± 0.7 years), authors reported maximal fat oxidation rates occurring at an average speed of 4.35 km·h^−1^ and corresponding to an intensity of 57.2% HR_max_ (Özdemir et al., 2019). Assuming an age-predicted maximal heart rate (i.e., 220–age) of 197.5 beats·min^−1^ for the present sample, it is possible that their PWS corresponded closely to predicted intensities (i.e., approximately 53% HR_max_). This speculation cannot be confirmed from the present data given that no maximal testing was performed to accurately determine maximal capacity. Fat oxidation could be a desired process for weight loss or maintenance, and walking at PWS could present advantages in that sense. While protocols of 3-min stages have been proven to be sufficient for determining maximal fat oxidation rates (Achten and Jeukendrup, 2004), walking at PWS for longer periods reveals contradictory findings as to the sustainability of maximum fat oxidation state. For example, Özdemir et al. (2019) found that the maximal rate of fat oxidation during walking decreases in the first 16 min of exercise, however, earlier studies have demonstrated an increased contribution of fat to energy production with longer exercise durations (Klein et al., 1994; Ravussin et al., 1986). In an effort to make exercise recommendations for sedentary individuals, duration needs to be considered given that the present study only reports short-term acute responses to walking.

The examination of spatiotemporal and gait stability parameters offers further insights into determinants of PWS. In previous work, PWS was shown to correspond to a typical spatiotemporal organization of gait in which a specific combination of stride frequency (SF) and stride length (SL) is adopted (Saibene and Minetti, 2003). Additionally, SF seems to play an important role in selection of PWS, as the U-shaped curve found between SF and metabolic cost of transport indicates an optimum at the preferred SF in both adults and children (Holt, Hamill and Andres, 1991; Jeng, Liao, Lai and Hou, 1997). Optimal temporal characteristics related to stride frequency were successfully mathematically predicted based on the resonant period of a force-driven harmonic oscillator (Holt, Hamill and Andres, 1990). According to these authors, walking at the preferred SF requires less muscle forces to maintain gait and potentially less mechanical work by body segments. Our results do not show an optimum in SL or SF around PWS *per se*, as both parameters increased significantly at each speed increment. Nevertheless, it is important to note that the organization of spatiotemporal gait parameters presented significantly higher variability (i.e., coefficient of variation, CoV) at speeds below PWS. Variability in stride characteristics is considered an indirect measure of gait stability (Hamacher et al., 2011; Fischer et al., 2021). This indicates that walking slower than PWS was less stable, and stability increased with speed until PWS where no further changes were seen in most variables. Importantly, CoV of the support phase duration presented as the most appropriate factor in the search of a PWS determinant, as its quadratic relationship with speed indicates an optimum exactly at PWS (Figure 3). As noted by Jordan et al. (2007), walking at PWS presents an enhanced stability and reproducibility compared to walking slower or faster, and thus behaves like a stable attractor. Any change in walking speed from PWS would result in the loss of dynamic stability. The low CoV values found in this study (i.e., approximately 3%) correspond to those found in healthy adults (Beauchet et al., 2009; Fischer et al., 2021) and reflect the general stability of this well-learned and repetitive motor skill. Interestingly, when comparing factors influencing PWS, Fischer et al. (2021) reported significantly higher CoV of contact time (i.e., support phase duration) in patients with lung disease as compared to healthy control group. This brings further support to our finding on the importance of contact time in gait that is directly linked to stability and risk of falls (Fischer et al., 2021).

Gender differences seen in some physiological and metabolic variables (i.e., EE, ME, HR and $\dot{V}$ E) were not related to differences in speeds as both male and female groups had similar PWS values. It is safe to assume that anthropometric differences might have affected values that were not normalized to body mass (e.g., EE, $\dot{V}$ E). The gender-differences observed in heart rate could also be potentially due to a difference in stroke volume or aerobic capacity ( $\dot{V}O_{2}$ _max_), however this could not be verified in the present study.

In sum, Figure 3 offers a visual summary of findings with the best fit functions of all studied variables and indicates the calculated optimum for quadratic models. Most of the studied variables were best fitted with a quadratic model and the predicted optimum corresponded to PWS or was non-significantly different in some variables that were considered here as potential determinants of PWS. Namely, mechanical efficiency, fat and carbohydrate oxidation and stability parameters met the criteria, and it is believed that participants chose their PWS based on these factors. A strength of the present study was its sample size (N = 34) as compared to other similar studies that examined 11 (Jordan et al., 2007), 12 (Willis et al., 2005), 14 (Godsiff et al., 2018), or 23 participants (Fernández Menéndez et al., 2019). In addition, the simultaneous examination of metabolic, perceptual, spatiotemporal and stability parameters at an absolute speed range around the individual PWS is also innovative as it brings a new perspective to studying the relationship between studied variables and speed. To our knowledge, no previous studies have done that, and the closest studies have examined different speeds presented as a percentage of PWS which makes it difficult to relate to actual magnitude of change in speed when examining relationships with intensity. Moreover, this also makes it harder to recommend a walking exercise on treadmill. Findings allow us to predict for a sedentary young adult an average of 110 kcal spent in a 30-min walk at PWS. The best fit equations (Table 4) could be used to predict a further rise of 11 kcal for a 30-min walk at PWS+0.5 km·h^−1^ or an additional 43 kcal at PWS+1.5 km·h^−1^. The proportion of this energy expended coming from fat oxidation can also be predicted using the equations.

One weakness of the present study is that walking was performed on a treadmill rather than overground, and in a controlled laboratory environment. While most studies have used a treadmill to acquire larger number of gait cycles or due to other limitations (e.g., equipment), walking on a treadmill seems to be more stable and less variable (Dingwell et al., 2001). However, both treadmill and overground locomotion do not seem to present mechanical differences (van Ingen Schenau, 1980). Walking in a natural outdoor environment rather than a controlled laboratory one also confers additional psychological benefits that were not accounted for in the present study (Focht, 2009). Indeed, in an outdoor environment and as compared to a laboratory environment, preferred walking speed is expected to be higher and ratings of perceived exertion lower (Focht, 2009). Another weakness of the current study relates to the duration of speed trials that only allow prediction of short-term acute responses to walking. Even though a steady state was reached at each trial (given the rather low-intensity nature of all trials), potential changes in various responses with time are to be investigated in future studies. Furthermore, similar future study designs should include maximal exercise testing to account for maximal aerobic capacity and anaerobic thresholds, which could provide a better understanding of the relative effort and intensity reached at PWS, compared to lower and higher speeds. This would help to obtain insights into whether walking at PWS occurs within general physical activity intensity recommendations. Indeed, de Moura et al. (2011) have verified that most normal weight individuals adopt a PWS that falls into the “moderate” intensity classification and was judged adequate to elicit health benefits according to ACSM recommendations.

In sum, the present findings support benefits of walking at the preferred walking speed for sedentary young adults, as opposed to walking at a slower or faster prescribed intensity. The choice of the preferred walking speed seems to be determined by factors being optimized at that intensity and related to mechanical efficiency, stability of gait and fuel oxidation. Therefore, walking at one’s preferred intensity could be an effective strategy for young sedentary adults to become active. Given the increased fat oxidation at the preferred intensity, walking at PWS could also serve as a safe exercise for weight management or disease prevention. Future studies could focus on examining responses to preferred walking for different portions of healthy and clinical populations. Finally, it is recommended that physical activity guidelines focus less on prescribing intensities and more on encouraging self-determined active behaviors and intensities, not just for their long-term benefits on adherence and overall health, but also for their potential acute benefits even when performed in short bouts.

## Data Availability

The original contributions presented in the study are included in the article/Supplementary material, further inquiries can be directed to the corresponding author.
